# Associations between Awareness of the Risk of Exposure to Pollutants Occurring at Fire Scenes and Health Beliefs among Metropolitan Firefighters in the Republic of Korea

**DOI:** 10.3390/ijerph19148860

**Published:** 2022-07-21

**Authors:** Hyeeun Oh, Soojin Kim, Hyekyung Woo, Seunghon Ham

**Affiliations:** 1 Department of Health Administration, Kongju National University, Gongju 32588, Korea; ohe.2065@gmail.com; 2 Fire Science Research Center, Seoul Metropolitan Fire Service Academy, Seoul 03312, Korea; kdamian0@snu.ac.kr; 3 Institute of Health and Environment, Kongju National University, Gongju 32588, Korea; 4 Department of Occupational and Environmental Medicine, Gil Medical Center, Gachon University College of Medicine, Incheon 21565, Korea

**Keywords:** firefighter, personal protection equipment (PPE), self-contained breathing apparatus (SCBA), health belief model (HBM), fire scene, pollutant, job-related exposure, exposure risk awareness (ERA), association

## Abstract

Firefighters are repeatedly exposed to various pollutants that occur at fire scenes. There are three levels of exposure: primary exposure to pollutants, secondary exposure to pollutants on personal protective equipment (PPE), and tertiary exposure to contaminated fire stations and fire engines due to pollutants on PPE. Therefore, it is important for firefighters to be aware of the risk of exposure to pollutants and to practice health behaviors such as appropriate PPE management. No clear association has been established in the existing literature between firefighters’ risk perception level and their health beliefs about the health impact of awareness of exposure to hazardous substances at fire scenes. This study aims to evaluate the relationship between awareness of the exposure risk to primary, secondary, and tertiary pollutants and health beliefs. It was designed as a cross-sectional study, in which a web-based survey was conducted from 13 May to 31 May 2021. The analysis was conducted in 1940 firefighters working in the Seoul metropolis who agreed to participate in the research. Participants who perceived susceptibility were likely to be aware of the primary (adjusted odds ratio (AOR) = 2.10, 95% confidence interval (CI) 1.16–3.80), secondary (AOR = 2.77, 95% CI 1.77–4.32), and tertiary (AOR = 2.73, 95% CI 1.85–4.03) exposure risks. Participants who perceived barriers were unlikely to be aware of the risk of exposure to primary (AOR = 0.67, 95% CI 0.49–0.91), secondary (AOR = 0.77, 95% CI 0.61–0.96), and tertiary (AOR = 0.75, 95% CI 0.62–0.91) pollutants. Educational intervention is recommended to improve perceived susceptibility and awareness of the risk of exposure to pollutants and reduce perceived barriers. Consequently, educational intervention is expected to positively impact firefighters’ management of appropriate PPE. We confirmed an association between occupational exposure risk and firefighters’ health beliefs. In the health belief model (HBM), health beliefs that affect health behavior also affect awareness of the exposure risk level. Therefore, an intervention for health beliefs can also be used to raise job-related exposure risk awareness. Regular training on the health impacts of fire scenes is necessary for both newcomers and incumbents to enable firefighters to better recognize the risks of each occupational exposure level. Additionally, laws and regulations are necessary for the removal of harmful substances that contaminate PPE, such as self-contained breathing apparatus (SCBA), during exposure to a fire scene. Our research can be used as a basis for improving fire policies and education programs in the future.

## 1. Introduction

Firefighters are repeatedly exposed to various combustible compounds and toxic substances generated at fire scenes. Toxic substances such as benzo[a]pyrene (PAH), carbon monoxide (CO), formaldehyde (HCHO), hydrogen cyanide (HCN), organophosphate flame retardants (OPFRs), and polybrominated diphenyl ethers (PBDEs) are generated at a fire scene. In addition, toxic substances such as 1,3-butadiene, benzene, toluene, xylene, HCHO, hydrogen chloride, HCN, ammonia, CO, and nitric acid are generated during live fire training for the purpose of educating firefighters [1,2,3]. Consequently, firefighters are constantly and repeatedly exposed to all these environments. This occupational exposure increases the risk of cancer among firefighters. Occupational cancers that occur in firefighters include those of the bladder, brain, and CNS as well as colorectal cancers, non-Hodgkin’s lymphoma, skin melanoma, and prostate and testicular cancers. In 2022, occupational exposure as a firefighter was classified as “carcinogenic to humans” (Group 1) based on “sufficient” evidence for cancer in humans [4]. Since various hazardous substances from combustion at fire scenes are generated that are beyond the safe range, it is extremely important for firefighters to be aware of the risks of occupational exposure and to prevent occupational diseases by wearing personal protective equipment (PPE), such as fire-protective suits and self-contained breathing apparatuses (SCBAs) [5]. Direct exposure to hazardous substances generated at a fire scene is called primary contamination or direct exposure. In addition, exposure of PPE such as firefighters’ protective clothing and SCBA worn at the scene of a fire to contaminants from the surrounding environment is called cross (secondary) contamination or indirect exposure [6]. Finally, cross-contamination by pollutants from entering a fire vehicle or fire department without going through an on-scene emergency decontamination process is called tertiary (third) contamination or indirect exposure. Therefore, it is extremely important to urgently decontaminate PPE at a fire scene after firefighting activities to minimize indirect exposure. As these three stages of contamination and job-related exposure are possible factors in long-term health, it is crucial for firefighters to be aware of this fact and practice healthy behaviors. Risk awareness and health belief are considered to be among the major factors in health behavior that many studies have examined [7,8,9,10,11]. In particular, recognizing risk can affect health behaviors: studies have found that the higher the risk awareness level, the higher the probability of practicing healthy behaviors [12]. In addition, it has been confirmed that health beliefs are closely related to health behavior practices: the higher the health belief level, the higher the rate of health behavior practice [13].

Health beliefs refer to individual’s subjective beliefs that are the source of actions taken to prevent diseases [14]. They refer to actions taken by individuals who believe they are healthy to prevent or detect a specific disease in the absence of pathological clinical symptoms [15]. The health belief model (HBM) was applied in our study as a social psychological health behavior change model developed to explain and predict health-related behaviors [16]. Studies on the effects of health beliefs on health behaviors have been conducted in various fields and have theoretically confirmed these effects, including the associated HBMs [17,18,19,20]. Research on firefighters’ risk perceptions is scanty, while the associated factors have not been identified. Although it is difficult in the case of firefighters to clearly identify the causal relationship between the two factors, because no study has examined the impact of risk perception and health beliefs on health behavior during field activities, it is expected that there will be a correlation. For firefighters with a high risk of exposure to carcinogenic substances to work safely, it is important for them to recognize that firefighting scenes can be harmful to health in the long term; thus, research on the risk perception factors is required. The research on the HBM conducted for firefighters has focused on evaluating their use, cleaning, and management behavior [21]. However, no studies have been conducted on health beliefs regarding the risk of exposure to hazardous substances and awareness of health effects at fire scenes.

Therefore, this study aims to evaluate the relationship between the level of awareness of health effects and firefighters’ health beliefs about the risks of occupational exposure to hazardous substances generated at fire scenes in the Seoul metropolitan area.

## 2. Materials and Methods

### 2.1. Korean Firefighter’s SCBA Survey (KFSS)

In this questionnaire survey, we focused on the SCBA and SCBA charger among the PPE. By establishing problems in the fields of human resources, equipment, budget, and systems through the results of the questionnaire, we searched for improvements in fire scenes by field.

In the Fire Science Research Center, Seoul Metropolitan, Fire Service Academy, a questionnaire survey was conducted in seven major domains among people in charge of fire suppression and rescue operations working in the Seoul metropolis. The specific contents of the questionnaire survey were as follows:

(1) Risk awareness of the generation of hazardous substances at fire scenes and the level of occupational exposure;
(2) Storage, use, washing, charging, training, repair, management status, and recognition of the safety management manual of the SCBA set (tank and mask);
(3) Usage and management of the SCBA charger and SCBA charging room installation environment;
(4) The SCBA tank inspection and SCBA repair room operation system;
(5) Health belief in the use and management of the SCBA: perceived susceptibility, perceived severity, perceived benefits, perceived barriers, and self-efficacy;
(6) Exposure to fire incidence and dispatch status;
(7) Demographic and sociological information of questionnaire participants.

### 2.2. Study Setting

The Seoul Metropolitan Fire and Disaster Prevention Headquarters, located in the Seoul metropolis in the Republic of Korea, has 7196 firefighters working at 119 safety centers and 24 fire departments under one headquarter as of January 2021. According to the job character classification, personnel are distributed as follows: 1130 for fire administration (15.7%), 483 for disaster command (6.7%), 155 for emergency communication and operation (2.2%), 3083 for fire suppression and investigation (42.8%), 671 for rescue (9.3%), and 1428 for emergency medical service (19.8%). Regarding the form of work, all operations except administrative work use a shift work system. In particular, unlike in the United States, all Korean firefighters operate according to a career firefighter system. In Korea, when firefighting activities are completed at a fire scene, most firefighters return to the fire station without following the emergency decontamination procedures for PPE exposed to contaminants generated at the scene. In the case of emergency decontamination at a fire scene among bystanders, less than 10% follow the procedures. The reason is that the emergency decontamination process is not included in the scene evacuation stage of the standard disaster site operation procedure; thus, there are no procedural regulations. Eight fire schools nationwide are in charge of training courses for new hires and training for incumbent firefighters. In particular, education on the health risks of pollutants generated at fire scenes is not provided to all firefighters at the fire academy.

### 2.3. Study Design and Subjects

**Figure 1:**
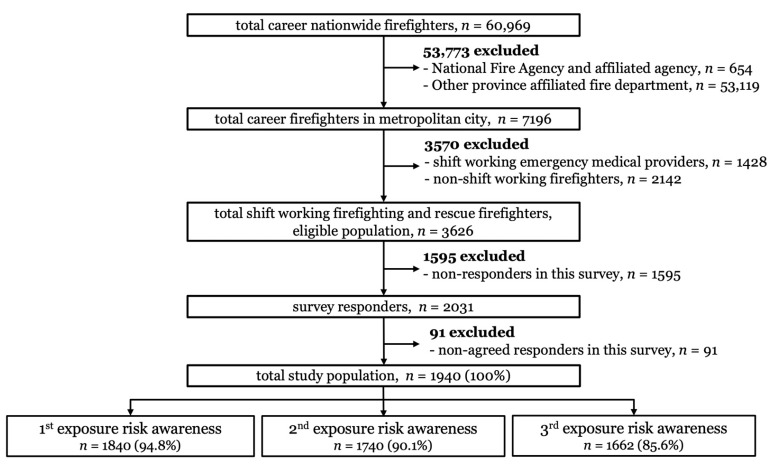
Inclusion criteria of study population in this study.

This study was cross-sectional in design. We conducted a questionnaire using the web-based Seoul Metropolitan Electronic Questionnaire System. As of May 2021, the survey was sent to 3626 shift workers engaged in fire and rescue operations, out of a total of 7196 career firefighters working in the Seoul metropolitan area. Of the 2031 survey respondents, only the 1940 who agreed to participate in the study and responded to the survey were analyzed (Figure 1). The questionnaire was completed via an electronic questionnaire system using a computer or cell phone. Firstly, we sent official text messages to the institutions where survey participants were employed, and secondly, we sent e-mails and SMS messages in order for the survey participants to actively participate. Consequently, at least 30% of respondent fire departments participated in the survey (Figure 1).

### 2.4. Data Collection

To investigate firefighters’ level of risk awareness about pollutants generated at fire scenes, whether they could affect health, and health beliefs about the use and management of respirators, we developed questionnaire items through prior research and interviews with experts in relevant fields. We evaluated the survey items through a pilot survey. The questionnaire survey was conducted for a total of 19 days, from 13 May to 31 May 2021. By explaining the purpose and importance of the questionnaire through official texts, e-mails, and SMS messages, we encouraged the survey participants to enroll. The main data collected by the questionnaire survey were demographic and sociological information (gender, age, job ranking, job duties, the total number of years of fire service, and the total number years of shift work in the fire service), information on the perception of the risk of occupational exposure level related to the occurrence of carcinogens at fire scenes and the perception of the possibility of health effects (recognition of risks of direct and indirect exposure to primary, secondary, and tertiary pollutants at fire scenes), and health beliefs about the use and management of SCBA (perceived susceptibility to occupationally related disease, perceived severity of occupationally related disease, perceived benefit from using SCBA, perceived barriers related to the firefighting service, and perceived self-efficacy. In particular, awareness of exposure to primary pollutants was defined as the recognition by firefighters themselves that toxic substances generated at fire scenes, including fires, explosions, and collapses, can cause cancer in the human body by absorption through breathing and the skin. Awareness of exposure to secondary pollutants was defined as the recognition that substances in PPE, including SCBA worn at fire scenes, can cause cancer in humans. Awareness of exposure to tertiary pollutants was defined as the recognition that pollutants can spread inside fire fighting vehicles and fire stations when firefighters board a fire engine and subsequently enter a fire station without having passed through emergency decontamination of the PPE worn at the fire scene.

### 2.5. Variables

The dependent variables were classified into three categories depending on the level of awareness of the risk of exposure to pollutants at a fire scene. The risk to health of direct exposure at the scene was primary; the risk of indirect exposure, such as through PPE and firefighting vehicles, was secondary; and the risk of exposure in the fire department was tertiary. The independent variable was health beliefs about firefighters’ use and management of SCBAs: perceived susceptibility, perceived severity, perceived benefits, perceived barriers, and perceived self-efficacy.

Covariates included the following demographic and job-related characteristics: gender, age, job ranking, job duties, first-time job duty, affiliation, the total number of years of firefighters’ working, the total number of years of shift work on fire and rescue duty, the monthly average number of fire dispatches, and the monthly average number of fire suppression cases in incomplete fires or more in the past year.

### 2.6. Health Belief Measures

We evaluated the firefighters’ health beliefs regarding the use of SCBA and the occurrence of occupational diseases. Health belief comprised 27 items in 5 domains: 8 items of perceived susceptibility, 7 items of perceived severity, 4 items of perceived benefit of using SCBA, 4 items of perceived barriers, and 4 items of perceived self-efficacy. The response items were measured on 5-point Likert-type scales. In responses to all the questions, 1 denoted “not at all,” while 5 denoted “very much.” The reliability of the items for each domain was measured by Cronbach’s alpha value. Perceived susceptibility measured the participants’ perceptions about developing an occupationally related disease in the future [22], while perceived severity measured their perceptions of the severity or impact that occupationally related disease could have on their lives in the future [22]. Perceived benefits measured the participants’ perceptions of benefits related to the proper use and management of SCBA. Perceived barriers measured their perceptions of barriers that would inhibit them from smoking cessation, lung function tests, and tests for job-related diseases. Self-efficacy measured their confidence in their abilities to properly use, test, store, repair, and manage SCBA.

### 2.7. Statistical Analysis

We performed a distribution of categorical variables reported as percentages and a $\chi^{2}$-test analysis to confirm the participants’ characteristics and differences between the dependent variables. We performed an exposure variable *t*-test to confirm the mean difference in health beliefs depending on exposure risk awareness of pollutants. To obtain the final model, we tested for interactions between exposure variables and potential covariates, assessed confounding factors, and performed precision level tests. The goodness of fit of the multivariable logistic regression model was evaluated using the Hosmer–Lemeshow (HL) test, while chi-square analysis for calibration performance was performed to assess the discrimination performance of the final models. All statistical analyses were conducted using the IBM SPSS 27.0 program (IBM Corporation: New York, NY, USA).

## 3. Results

### 3.1. Demographic Characteristics of Eligible Study Population by Job-Related Exposure Risk Awareness

From the table showing demographic characteristics according to firefighters’ awareness of exposure risk at fire scenes, most respondents were male (1886 persons, 97.2%), in their 50s or older (835 persons, 43.1%), and were fire lieutenants (974 persons, 50.2%) (Table 1). Most participants were in charge of fire suppression (1176 persons, 60.6%), had a total work period as firefighters of more than 25 years (608 persons, 31.3%), had a total fulfillment period of fire and rescue duty in the type of shift work for 5 to 14 years (667 persons, 34.4%), and were affiliated with 119 safety centers (1001 persons, 51.6%). The monthly average number of fires in the past year was from 10 to 14 for most participants (463 persons, 23.9%) while the monthly average number of fire suppression cases in incomplete fires or more in the past year was from 1 to 2 for most participants (513 persons, 26.4%).

Of the participants, 94.8% perceived primary exposure risk awareness (ERA) (1840 persons), 90.1% perceived secondary ERA (1747 persons), and 85.6% perceived tertiary ERA (1662 persons). All female participants perceived primary ERA (54 persons, 100%). Participants in their 50s or older had a lower perception rate for ERA than those in other age groups. Participants who had worked for more than 25 years had a lower ERA perception rate than other participants. Participants in charge of rescue had higher perception rates for primary (96.5%), secondary (93.4%), and tertiary (91.3%) ERA than those in charge of other assigned tasks.

**Figure 2:**
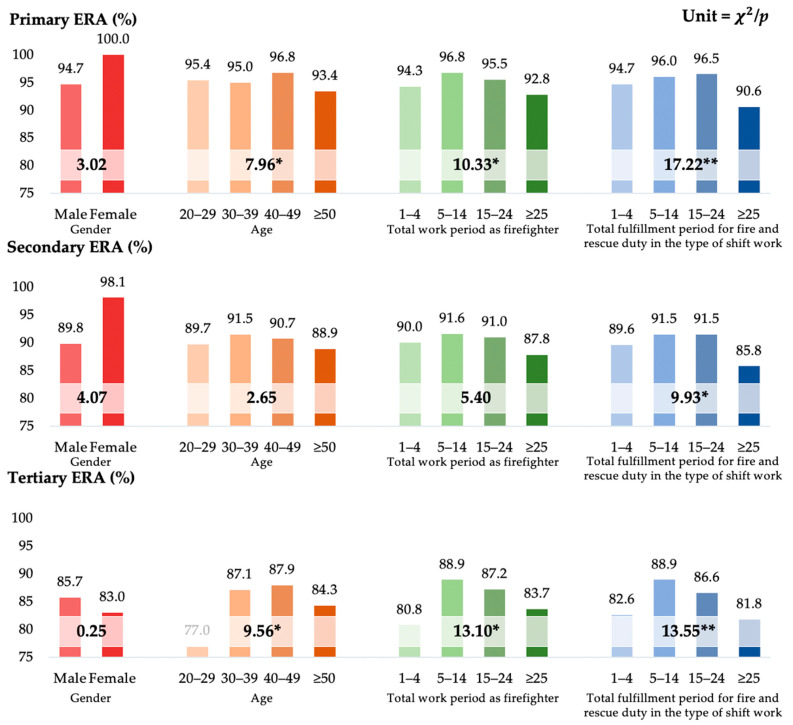
Differences in demographic characteristics by awareness of exposure risk to pollutants. ERA: exposure risk awareness; *: *p* < 0.05, **: *p* < 0.01.

Females had higher awareness rates for primary (100%) and secondary (98.1%) exposure risk than males; however, males had higher awareness rates for tertiary exposure risk (85.7%) (Figure 2). Participants who had worked for 5–14 years as firefighters had the highest awareness rates for risk exposure to all pollutants (primary ERA = 96.8%, secondary ERA = 91.6%, and tertiary ERA = 88.9%), and those who had worked for more than 25 years generally had the lowest (primary ERA: 92.8%, secondary ERA: 87.8%, and tertiary ERA: 83.7%). Participants with a total fulfillment period for fire and rescue duty in the type of shift work of more than 25 years had the lowest awareness rates for risk exposure to all pollutants (primary ERA: 90.6%, secondary ERA: 85.8%, and tertiary ERA: 81.8%).

There were differences in the primary (*p* < 0.05) and tertiary (*p* < 0.01) ERA according to age and total work period as firefighter, and there were differences in the primary (*p* < 0.01), secondary (*p* < 0.05), and tertiary (*p* < 0.01) ERA according to the total fulfillment period for fire and rescue duty in the type of shift work. There was no difference in the secondary ERA.

### 3.2. Health Beliefs among Firefighters

Table 2 shows the mean difference in health beliefs depending on whether participants perceived ERA. Participants who perceived ERA, regardless of the level of ERA, had a higher mean of perceived susceptibility, perceived severity, perceived benefits, and self-efficacy than those who did not. The mean difference in health beliefs between participants who perceived ERA and those who did not was the largest in primary ERA and the smallest in tertiary ERA.

Participants who perceived primary ERA had higher susceptibility (3.96 ± 0.62), severity (4.18 ± 0.66), benefits (4.20 ± 0.68), and self-efficacy (3.54 ± 0.71) than those who did not, while barriers (2.47 ± 0.76) were lower than for those who did not. Participants who perceived secondary ERA had higher susceptibility (3.98 ± 0.62), severity (4.20 ± 0.67), benefits (4.21 ± 0.68), and self-efficacy (3.55 ± 0.72) and lower barriers (2.47 ± 0.76) than those who did not. Participants who perceived tertiary ERA had higher susceptibility (4.00 ± 0.62), severity (4.21 ± 0.67), benefits (4.23 ± 0.68), and self-efficacy (3.56 ± 0.72) and lower barriers (2.45 ± 0.77) than those who did not. There were significant differences overall.

#### 3.2.1. Perceived Susceptibility to Occupationally Related Diseases

The questionnaire on perceived susceptibility to work-related diseases comprised a total of eight items. Of the participating firefighters, 58.9% (*n* = 1143) agreed or strongly agreed with, “I know the risk of disease related to the job of a firefighter”; 38.8% (*n* = 752) agreed or strongly agreed with, “I have seen a colleague firefighter being diagnosed with a firefighting service-related disease”; 84.9% (*n* = 1648) agreed or strongly agreed with, “I think you can get sick if you do not wear an SCBA completely at the fire scene”; 83.9% (*n* = 1628) agreed or strongly agreed with, “I think that firefighters who have been exposed to much firefighting (including investigation and identification) and lifesaving work at fire scenes are more likely to contract occupational diseases than if they had not been”; 75.9% (*n* = 1472) agreed or strongly agreed with, “I think the inside of the vehicle may be contaminated with toxic substances when an SCBA that has not been used in the field for education and training is loaded in a personal car”; 62.2% (*n* = 1207) agreed or strongly agreed with, “I am worried about contracting an occupational disease related to firefighting work”; 72.7% (*n* = 1410) agreed or strongly agreed with, “I think I can also contract occupational diseases whenever I hear stories or news about firefighting-related occupational diseases”; and 79.5% (*n* = 1542) agreed or strongly agreed with, “I know that wearing an SCBA properly reduces the risk of occupational diseases even during live fire trainings.” Cronbach’s alpha value for the 8-item perceived susceptibility questionnaire was 0.88.

#### 3.2.2. Perceived Severity of Occupationally Related Diseases

The questionnaire on perceived severity of work-related diseases comprised a total of seven items. Of the participating firefighters, 82.7% (*n* = 1604) agreed or strongly agreed with, “I know that firefighting work can cause respiratory diseases”; 75.1% (*n* = 1457) agreed or strongly agreed with, “I know about the effects of exposure to firefighting sites on health”; 72.8% (*n* = 1413) agreed or strongly agreed with, “I am aware of the effects of disease incidences related to my work on work performance and daily activities”; 82.9% (*n* = 1628) agreed or strongly agreed that “If you get sick from your job, your daily life will be limited”; 85.3% (*n* = 1654) agreed or strongly agreed with, “Occupational diseases that can be acquired by firefighters can reduce the quality of life”; 80.7% (*n* = 1565) agreed or strongly agreed with, “Occupational diseases that can be contracted from firefighters are diseases with high mortality”; and 85.0% (*n* = 1649) agreed or strongly agreed with, “Occupational diseases that can be contracted from firefighters are highly related to the working environment.” Cronbach’s alpha value for the seven-item perceived sensitivity questionnaire was 0.95.

#### 3.2.3. Perceived Benefits

The questionnaire on perceived benefits comprised a total of four items. Of the participants, 77.4% (*n* = 1501) agreed or strongly agreed with, “SCBA worn according to the standards during live fire training helps to prevent occupational diseases”; 82.5% (*n* = 1600) agreed or strongly agreed with, “I know that the correct use of an SCBA ensures my safety and reduces health risks at the firefighting scene”; 82.5% (*n* = 1619) agreed or strongly agreed with, “Proper use and management of SCBA helps to reduce occupational exposure in firefighting work and maintains safety”; and 86.7% (*n* = 1682) agreed or strongly agreed with, “Prevention of possible diseases or accidents is very important for my health.” Cronbach’s alpha value for the four perceived benefits was 0.94.

#### 3.2.4. Perceived Barriers

The questionnaire on perceived barriers comprised a total of four items. Of the respondents, 24.8% (*n* = 481) agreed or strongly agreed with, “It is cumbersome to undergo a pulmonary function test,” while 45.2% (*n* = 876) responded negatively or strongly disagreed; 15.9% (*n* = 308) responded with, “I do not know how to arrange for a pulmonary function test” or strongly agreed, and 56.1% (*n* = 1088) disagreed or strongly disagreed. To the question, “It is difficult for me to quit smoking,” 19.0% (*n* = 369) of the respondents agreed or strongly agreed, and 59.6% (*n* = 1157) disagreed or strongly disagreed. Of the respondents, 19.3% (*n* = 375) agreed or strongly agreed with, “I am not interested in occupational diseases related to firefighting work,” and 42.0% (*n* = 814) disagreed or strongly disagreed. Cronbach’s alpha value for the four perceived benefit questions was 0.59.

#### 3.2.5. Perceived Self-Efficacy

The questionnaire on perceived self-efficacy comprised a total of four items. Of the respondents, 70.1% (*n* = 1359) agreed or strongly agreed with, “I can use an SCBA correctly”; 60.2% (*n* = 1167) agreed or strongly agreed with, “I can properly inspect the SCBA”; 54.1% (*n* = 1050) agreed or strongly agreed with, “I can store the SCBA as recommended”; and 24.5% (*n* = 475) agreed or strongly agreed with, “I can properly repair the SCBA.” Cronbach’s alpha value for the 4-item perceived self-efficacy questionnaire was 0.83.

### 3.3. Regression of HBM Constructs

Table 3 shows how each health belief affected primary, secondary, and tertiary ERA. In particular, perceived susceptibility and perceived barriers were clearly related to awareness of the risk of exposure to pollutants. Perceived severity was associated with primary ERA, but not with secondary and tertiary ERA. The adjusted odds ratio (AOR) values for perceived benefits and self-efficacy increased but were not statistically significant.

Participants who perceived susceptibility were more likely to be aware of primary (AOR = 2.10, 95% CI 1.16–3.80), secondary (AOR = 2.77, 95% CI 1.77–4.32), and tertiary (AOR = 2.73, 95% CI 1.85–4.03) exposure risk. Participants who perceived severity were more likely to be aware of primary exposure risk (AOR = 3.06, 95% CI 1.49–6.27); however, this association was not significant for secondary (AOR = 1.42, 95% CI 0.83–2.40) and tertiary (AOR = 1.23, 95% CI 0.78–1.94) exposure risk. Participants who perceived barriers were more unlikely to be aware of primary (AOR = 0.67, 95% CI 0.49–0.91), secondary (AOR = 0.77, 95% CI 0.61–0.96), and tertiary (AOR = 0.75, 95% CI 0.62–0.91) exposure risk.

## 4. Discussion

### 4.1. Implications for Awareness of Occupational Exposure Risk and Importance of Health Behavior

This study evaluated the relationship between awareness of the risk of exposure to pollutants that occur at fire scenes and health beliefs by metropolitan firefighters in the Republic of Korea. To clarify the relationship, a web-based survey was conducted among firefighters in the Republic of Korea. The results of the study indicated a distinct relationship between awareness of the risk of exposure to pollutants and health beliefs. In particular, awareness of the risk of exposure to pollutants was evidently associated with perceived susceptibility and perceived barriers. In a previous study [23], firefighters were found to exhibit a high level of perceived susceptibility, consistent with the results of this research. Other previous studies [24] found an extremely high level of perceived severity among firefighters, whereas a generally high level was found in this study. Although the relationship between health behavior and health beliefs has been corroborated in previous studies, such as proper PPE use, management, and cleaning, insufficient studies have evaluated the relationship between awareness of the risk of exposure to pollutants and health beliefs [21].

We found differences in awareness of the risk of exposure to pollutants based on demographic characteristics. Among the participants who were aware of the risk of exposure to pollutants, those in charge of rescue had a higher awareness rate than those in charge of other tasks, including fire suppression (Table 1). These results were consistent with those of previous studies that rescue workers showed the most awareness for harmful factors among the task categories [25]. One explanation that has been offered is that rescue workers’ awareness rate for the risk of exposure to pollutants is high because they work closest to various dangerous sites, including fires [25]. Indeed, it was found to be statistically significant that firefighters in charge of rescue had a higher risk of exposure to harmful substances such as dust, organic solvents, and other chemicals than workers in other task categories [26]. It is suggested that the risk of exposure to pollutants requires intervention first in order for the participants in charge of rescue to practice healthy behaviors to prevent diseases.

In this study, there were differences in the primary, secondary and tertiary ERA according to the total fulfillment period for fire and rescue duty in the type of shift work. However, for participants with relatively long total work periods as firefighters or total fulfillment period as firefighters and rescue workers in their types of shift work, the awareness rate for the risk of exposure to pollutants was rather low. According to previous studies, various programs, including motivational and mental health education programs, were developed for new firefighters with short work periods or shift periods as firefighters [27,28]. However, intervention for firefighters with relatively long total work periods or total fulfillment periods for shift work is insufficient. Indeed, after basic training for new firefighters, there are few regular firefighting-related educational programs for incumbents in the curriculum of the National Fire Academy and Provincial Fire Academy in Korea. As a program for incumbent firefighters, education is intended to improve on-scene safety and health awareness to train firefighters in charge of safety and reserve personnel; however, there are very few staff. Therefore, an intervention program to improve occupational safety and health awareness targeting incumbent firefighters is crucial. This could be an appropriate intervention program for firefighters with long years of service in the fire service. A study, [29], suggests that to attain an optimal level of risk perception, it is necessary to strengthen firefighters’ capabilities and efficacy through specialized firefighting education and training, and for this, a systematic response manual should be established.

In the HBM, it is theoretically unambiguous that health beliefs affect health behavior [30]. If health beliefs affect awareness of exposure risk as well as health behavior, intervention for health beliefs could also be used as an intervention for awareness of exposure risk [16]. We found that perceived susceptibility and perceived barriers, among health beliefs, affected awareness of the risk of exposure to direct and indirect pollutants, and that perceived severity affected awareness of the risk of exposure to direct pollutants. Therefore, the intervention n health beliefs could also be used on awareness of exposure risk. According to the HBM, it would ultimately have a positive effect on health behavior.

Perceived susceptibility is a subjective perception of the possibility of developing a disease and refers to the degree to which individuals feel about how exposed they are to the disease [16]. In this research, when susceptibility was perceived, there was a high likelihood of perceiving the risk of exposure to primary, secondary, and tertiary pollutants. According to previous studies, it is possible to determine whether perceived susceptibility can be practiced as a health behavior based on the degree of susceptibility [16]. It has also been suggested that perceived susceptibility can be increased through correct knowledge and educational intervention [31]. Educational intervention to increase perceived susceptibility would also increase the likelihood of perceiving the risk of exposure to pollutants, which could lead to the practice of healthy behavior. Therefore, it is necessary to develop an education program to improve firefighters’ awareness of the risk of exposure to pollutants.

It is theoretically evident that perceived barriers are the perception of the negative aspects of performing health behaviors, and that a high level of perceived barriers negatively affects health behavior [16]. In this study, those who perceived barriers were less likely to be aware of the risk of exposure to primary, secondary, and tertiary pollutants than those who did not. According to previous studies, participants’ perceived barriers significantly decreased after educational intervention [32]. Educational intervention to reduce perceived barriers will increase the likelihood of perceiving the risk of exposure to pollutants, which can lead to the practice of healthy behavior. Therefore, educational intervention is required to improve firefighters’ awareness of the risk of exposure to pollutants.

Perceived severity refers to how serious one feels about a disease and their belief in the sequelae. Perceived severity has been called a “perceived threat” with perceived susceptibility [16]. It has been suggested that high severity is necessary before perceived susceptibility becomes a strong behavior predictor, and that susceptibility and severity are related [16]. Therefore, there is a need to improve perceived severity for healthy behavior. Considering that perceived severity in this study demonstrated a clear association with direct contamination, awareness of the risk of exposure to indirect pollutants would increase further when mediated by perceived susceptibility. According to previous studies, specifying the importance of risk and health status has been suggested as not only important, but also a way to preferentially mediate awareness of the risk of exposure to indirect pollutants [16].

In this study, consistent results were found with respect to firefighters’ awareness of the direct (primary) and indirect (secondary and tertiary) risk of exposure to pollutants. In Table 1, the awareness rates for secondary and tertiary risks of exposure to pollutants are relatively lower than that for the primary risk of exposure risk to pollutants. In Table 2, the differences in health beliefs based on the secondary and tertiary risks of exposure to pollutants are smaller than those based on the primary risk of exposure to pollutants. Table 3 shows that there was a high likelihood of perceiving the primary risk of exposure to pollutants in the presence of perceived severity; however, the association was not significant in the case of awareness of the secondary and tertiary risks of exposure to pollutants. According to previous studies, PPE that retain some pollutants may contribute to firefighters’ systemic dose (secondary exposure). When PPE is doffed, often after the removal of SCBA, dry contaminants can become airborne and be inhaled. These studies also highlight the potential for take-home exposure (tertiary exposure) [33]. However, it has been suggested that proper PPE management is an effective way for firefighters to reduce the risk of secondary exposure to pollutants [6]. Thus, indirect exposure risk due to PPE affects not only individuals but also acquaintances (work colleagues, family, etc.). Therefore, awareness of indirect exposure is extremely important and necessitates the practice of healthy behaviors such as proper PPE management. Consequently, it is suggested that intervention in the awareness of indirect exposure risk is required first. It has been suggested that the expansion of safety devices at risk sites and the provision of active education and incentives for workers in dangerous environments is intervention for awareness of the risk of exposure to pollutants [25]. This intervention could ultimately be used to lead to healthy behavior such as proper PPE management and use.

### 4.2. Implications for Intervention in Occupational Health Practice

In Korea, if one passes the firefighter recruitment examination, they are hired after completing basic education at the fire academy. The curriculum for becoming a firefighter is divided into theory and practice and includes various fields such as fire, rescue, first aid, fire administration, disaster, and emergency communication. However, in the training course for firefighters who are in charge of and provide safety services to citizens, the training time for improving their safety and health awareness is not sufficient and needs to be increased.

Generally, when firefighting activities are completed at a fire scene, most firefighters return to the fire station without following emergency decontamination procedures for PPE exposed to pollutants generated at the scene. In previous studies, emergency decontamination was performed in less than 10% of fire scenes [34]. There are several reasons. First, there are no procedural restrictions as the emergency decontamination process is not included in the site evacuation stage among the standard fire scene operation procedures. Second, fire-scene decontamination procedures are practiced only in part of the training for new hires in the fire academy curriculum, while there is no fire suppression and emergency decontamination training course for incumbent firefighters. Repeated training for incumbents is urgently required. This is also shown by our study results. Third, although it is recognized that on-scene emergency decontamination is necessary, it is not practiced; thus, interventions that can lead to action are required. Eight fire academies across the country are responsible for training firefighters; in particular, education on the health risks of hazardous substances generated at fire scenes is lacking. Therefore, it is necessary to organize courses that emphasize the health effects of direct or indirect occupational exposure that occurs at fire scenes for new and incumbent firefighters. This is because early learning and repeated education can lead to self-protection thoughts and safety actions.

### 4.3. Strengths, Limitations and Further Studies

Our study is significant as it is the first to evaluate the level of awareness of firefighters’ own occupationally related exposure levels to toxic substances generated at fire scenes. It is also the first study to evaluate the level of risk awareness regarding an individual’s health beliefs. There are limited published studies on firefighters’ health beliefs [21,35]. In our study, we analyzed the relationship between awareness of the risk of exposure and health beliefs and found a strong association; thus, the need for intervention was confirmed.

We conducted research among firefighters in a metropolis, and all fire departments produced a survey participation rate of 30% or more, indicating representative data. However, concerning firefighters across the country, the fire culture, resources, and policies in each region are different; thus, our research results cannot be generalized and applied. In the future, it is necessary to expand the survey nationwide using the same survey tool as in the present study to enable different suggestions of intervention policies for different regions.

In this study, the analysis of health beliefs was limited to firefighters in charge of fire suppression, investigation, rescue, and safety work at fire scenes. In the future, paramedics in charge of emergency medical services will be included to investigate the relationship between occupational exposure factors and health beliefs.

Finally, as ours was a retrospective cross-sectional study, temporal relationships could not be established, and therefore causality could not be confirmed.

## 5. Conclusions

We confirmed an association between occupational exposure risk and the health beliefs of firefighters in charge of safety at fire scenes. Awareness of the level of risk of exposure to pollutants was associated with perceived susceptibility and perceived barriers. In particular, it was confirmed that rescuers were more aware of the risk of exposure to pollutants than other firefighter groups. Because this is the deepest way to save lives at a fire scene, it is known through experience in firefighting that there is a lot of exposure at the fire scene, and the awareness of the risk of exposure is inevitably high, which is linked to health behavior. As such, health beliefs that influence health behaviors in HBM also influence awareness of occupational exposure risk levels. Therefore, intervention for health beliefs can also be used for raising job-related exposure risk awareness.

As the result of this study, recognizing that the risk of exposure to pollutants generated at a fire scene can affect health requires a priority intervention so that firefighters can practice health behaviors to prevent occupational diseases. Therefore, it is necessary to repeatedly conduct training on the health impacts of fire scenes for both newcomers and incumbents so that firefighters can better recognize the risks of each occupational exposure level. In addition, it is necessary to enact laws and regulations that enable the removal of harmful substances that contaminate PPE, such as SCBA, during exposure to fire scenes. Our research can be used as a basis for improving fire policies and education programs in the future.

## Figures and Tables

**Table 1:** Demographic characteristics of the level of job-related exposure risk awareness among firefighters. Unit: N (%).

Potential Risk Factors		Total	Primary ERA		Secondary ERA		Tertiary ERA	
			Yes	No	Yes	No	Yes	No
Gender								
	Male	1886 (97.2)	1786 (97.1)	100 (100.0)	1694 (97.0)	192 (99.5)	1617 (97.3)	269 (96.8)
	Female	54 (2.8)	54 (2.9)	0 (0.0)	53 (3.0)	1 (0.5)	45 (2.7)	9 (3.2)
Age								
	20–29	87 (4.5)	83 (4.5)	4 (4.0)	78 (4.5)	9 (4.7)	67 (4.0)	20 (7.2)
	30–39	480 (24.7)	456 (24.8)	24 (24.0)	439 (25.1)	41 (21.2)	418 (25.2)	62 (22.3)
	40–49	538 (27.7)	521 (28.3)	17 (17.0)	488 (27.9)	50 (25.9)	473 (28.5)	65 (23.4)
	≥50	835 (43.1)	780 (42.4)	55 (55.0)	742 (42.5)	93 (48.2)	704 (42.4)	131 (47.1)
Job Ranking								
	Firefighter	228 (11.7)	217 (11.8)	11 (11.0)	204 (11.7)	24 (12.4)	184 (11.1)	44 (15.8)
	Senior Firefighter	310 (16.0)	296 (16.1)	14 (14.0)	280 (16.0)	30 (15.5)	271 (16.3)	39 (14.0)
	Fire Sergeant	362 (18.7)	352 (19.1)	10 (10.0)	339 (19.4)	23 (11.9)	328 (19.7)	34 (12.2)
	Fire Lieutenant	974 (50.2)	912 (49.6)	62 (62.0)	864 (49.5)	110 (57.0)	818 (49.2)	156 (56.1)
	Over Fire Captain	66 (3.4)	63 (3.4)	3 (3.0)	60 (3.4)	6 (3.1)	61 (3.7)	5 (1.8)
Job Duty								
	Firefighter	1176 (60.6)	1119 (60.8)	57 (57.0)	1046 (59.9)	130 (67.4)	988 (59.4)	188 (67.6)
	Rescue	288 (14.8)	278 (15.1)	10 (10.0)	269 (15.4)	19 (9.8)	263 (15.8)	25 (9.0)
	Others	476 (24.5)	443 (24.1)	33 (33.0)	432 (24.7)	44 (22.8)	411 (24.7)	65 (23.4)
First-Time Job Duty								
	Firefighter	1260 (64.9)	1206 (65.5)	54 (54.0)	1133 (64.9)	127 (65.8)	1073 (64.6)	187 (67.3)
	Fire Investigator	6 (0.3)	5 (0.3)	1 (1.0)	5 (0.3)	1 (0.5)	5 (0.3)	1 (0.4)
	Fire Prevention	17 (0.9)	16 (0.9)	1 (1.0)	16 (0.9)	1 (0.5)	15 (0.9)	2 (0.7)
	Rescue	246 (12.7)	238 (12.9)	8 (8.0)	230 (13.2)	16 (8.3)	225 (13.5)	21 (7.6)
	EMT	74 (3.8)	71 (3.9)	3 (3.0)	66 (3.8)	8 (4.1)	59 (3.5)	15 (5.4)
	Fire Engine Driver	337 (17.4)	304 (16.5)	33 (33.0)	297 (17.0)	40 (20.7)	285 (17.1)	52 (18.7)
Total Work Period as Firefighter (year)								
	1–4	28 1(14.5)	265 (14.4)	16 (16.0)	253 (14.5)	28 (14.5)	227 (13.7)	54 (19.4)
	5–14	560 (28.9)	542 (29.5)	18 (18.0)	513 (29.4)	47 (24.4)	498 (30.0)	62 (22.3)
	15–24	491 (25.3)	469 (25.5)	22 (22.0)	447 (25.6)	44 (22.8)	428 (25.8)	63 (22.7)
	≥25	608 (31.3)	564 (30.7)	44 (44.0)	534 (30.6)	74 (38.3)	509 (30.6)	99 (35.6)
Total Fulfillment Period for Fire and Rescue Duty in the Type of Shift Work (year)								
	1–4	413 (21.3)	391 (21.3)	22 (22.0)	370 (21.2)	43 (22.3)	341 (20.5)	72 (25.9)
	5–14	667 (34.4)	640 (34.8)	27 (27.0)	610 (34.9)	57 (29.5)	593 (35.7)	74 (26.6)
	15–24	508 (26.2)	490 (26.6)	18 (18.0)	465 (26.6)	43 (22.3)	440 (26.5)	68 (24.5)
	≥25	352 (18.1)	319 (17.3)	33 (33.0)	302 (17.3)	50 (25.9)	288 (17.3)	64 (23.0)
Affiliation								
	Field Operations Unit	853 (44.0)	807 (43.9)	46 (46.0)	775 (44.4)	78 (40.4)	745 (44.8)	108 (38.8)
	119 Safety Center	1001 (51.6)	951 (51.7)	50 (50.0)	894 (51.2)	107 (55.4)	840 (50.5)	161 (57.9)
	Special Rescue Unit	76 (3.9)	73 (4.0)	3 (3.0)	69 (3.9)	7 (3.6)	68 (4.1)	8 (2.9)
	Fire Service Academy	10 (0.5)	9 (0.5)	1 (1.0)	9 (0.5)	1 (0.5)	9 (0.5)	1 (0.4)
Monthly Average Number of Fires in the Past Year (case)								
	1–4	217 (11.2)	207 (11.3)	10 (10.0)	200 (11.4)	17 (8.8)	191 (11.5)	26 (9.4)
	5–9	408 (21.0)	390 (21.2)	18 (18.0)	367 (21.0)	41 (2.2)	343 (20.6)	65 (23.4)
	10–14	463 (23.9)	435 (23.6)	28 (28.0)	412 (23.6)	51 (26.4)	389 (23.4)	74 (26.6)
	15–19	260 (13.4)	251 (13.6)	9 (9.0)	239 (13.7)	21 (10.9)	222 (13.4)	38 (13.7)
	20–24	136 (7.0)	129 (7.0)	7 (7.0)	122 (7.0)	14 (7.3)	122 (7.3)	14 (5.0)
	≥25	456 (23.5)	428 (23.3)	28 (28.0)	407 (23.3)	49 (25.4)	395 (23.8)	61 (21.9)
Monthly Average Number of Fire Suppression Cases in Incomplete Fires or more in the Past Year (case)								
	None	118 (6.1)	113 (6.1)	5 (5.0)	104 (6.0)	14 (7.3)	99 (6.0)	19 (6.8)
	1–2	513 (26.4)	490 (26.6)	23 (23.0)	459 (26.3)	54 (28.0)	429 (25.8)	84 (30.2)
	3–4	477 (24.6)	452 (24.6)	25 (25.0)	428 (24.5)	49 (25.4)	413 (24.8)	64 (23.0)
	5–7	287 (14.8)	272 (14.8)	15 (15.0)	265 (15.2)	22 (11.4)	248 (14.9)	39 (14.0)
	8–9	157 (8.1)	145 (7.9)	12 (12.0)	139 (8.0)	18 (9.3)	130 (7.8)	27 (9.7)
	10–14	130 (6.7)	122 (6.6)	8 (8.0)	113 (6.5)	17 (8.8)	113 (6.8)	17 (6.1)
	≥15	258 (13.3)	246 (13.4)	12 (12.0)	239 (13.7)	19 (9.8)	230 (13.8)	28 (10.1)
**Total**		1940 (100)	1840 (100)	100 (100)	1747 (100)	193 (100)	1662 (100)	278 (100)

ERA: exposure risk awareness. EMT: emergency medical technician.

**Table 2:** Difference in health beliefs according to the level of awareness of the risk of exposure to pollutants.

	Mean SD			Mean Difference	
	Yes	No	*t*	MD (95% CI)	
	**Primary ERA**				
Perceived susceptibility	3.96 ± 0.62	3.31 ± 0.77	8.34 ***	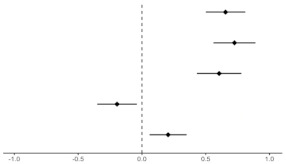	0.65(0.50, 0.81)
Perceived severity	4.18 ± 0.66	3.46 ± 0.82	8.62 ***		0.72(0.56, 0.89)
Perceived benefits	4.20 ± 0.68	3.60 ± 0.85	6.97 ***		0.61(0.43, 0.78)
Perceived barriers	2.47 ± 0.76	2.67 ± 0.75	−2.54 *		−0.2(−0.35, −0.04)
Self-efficacy	3.54 ± 0.71	3.34 ± 0.75	2.64 **		0.2(0.06, 0.35)
	**Secondary ERA**				
Perceived susceptibility	3.98 ± 0.62	3.44 ± 0.61	11.49 ***	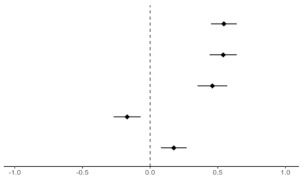	0.54(0.45, 0.64)
Perceived severity	4.20 ± 0.67	3.66 ± 0.70	10.52 ***		0.54(0.44, 0.64)
Perceived benefits	4.21 ± 0.68	3.76 ± 0.74	8.26 ***		0.46(0.35, 0.57)
Perceived barriers	2.47 ± 0.76	2.63 ± 0.65	−3.31 **		−0.17(−0.27, −0.07)
Self-efficacy	3.55 ± 0.72	3.37 ± 0.63	3.68 ***		0.18(0.08, 0.27)
	**Tertiary ERA**				
Perceived susceptibility	4.00 ± 0.62	3.49 ± 0.63	12.79 ***	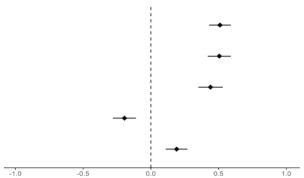	0.51(0.43, 0.59)
Perceived severity	4.21 ± 0.67	3.71 ± 0.68	11.56 ***		0.5(0.42, 0.59)
Perceived benefits	4.23 ± 0.68	3.79 ± 0.73	9.86 ***		0.44(0.35, 0.53)
Perceived barriers	2.45 ± 0.77	2.65 ± 0.65	−4.42 ***		−0.19(−0.28, −0.11)
Self-efficacy	3.56 ± 0.72	3.37 ± 0.63	4.56 ***		0.19(0.11, 0.27)

SD: standard deviation; MD: mean difference; CI: confidence interval; ERA: exposure risk awareness; *: *p* < 0.05, **: *p* < 0.01, ***: *p* < 0.001.

**Table 3:** Multiple logistic regression analysis of awareness of the risk of exposure to pollutant factors and health belief constructs.

	Primary ERA	Secondary ERA	Tertiary ERA
	AOR (95% CI)	AOR (95% CI)	AOR (95% CI)
Health Beliefs			
Perceived Susceptibility	2.10 (1.16–3.80)	2.77 (1.77–4.32)	2.73 (1.85–4.03)
Perceived Severity	3.06 (1.49–6.27)	1.42 (0.83–2.40)	1.23 (0.78–1.94)
Perceived Benefits	0.79 (0.45–1.36)	0.95 (0.64–1.43)	1.06 (0.75–1.49)
Perceived Barriers	0.67 (0.49–0.91)	0.77 (0.61–0.96)	0.75 (0.62–0.91)
Self-Efficacy	0.67 (0.45–1.00)	0.85 (0.65–1.12)	0.93 (0.73–1.17)
Hosmer and Lemeshow χ^2^	6.065	6.727	13.918
*p*-value	0.640	0.566	0.084

ERA: exposure risk awareness; AOR: adjusted odds ratio; CI: confidence interval. Adjusted variables: age, job ranking, and job duty.

## Data Availability

The data that support the findings of this study are available from the corresponding author upon reasonable request.
